# Childhood pneumonia and meningitis in the Eastern Highlands Province, Papua New Guinea in the era of conjugate vaccines: study methods and challenges

**DOI:** 10.1186/s41479-017-0029-y

**Published:** 2017-03-05

**Authors:** Christopher C. Blyth, Rebecca Ford, Joycelyn Sapura, Tonny Kumani, Geraldine Masiria, John Kave, Lapule Yuasi, Andrew Greenhill, Ilomo Hwaihwanje, Amanda Lang, Deborah Lehmann, William Pomat, on behalf of the Papua New Guinea Pneumonia and Meningitis Etiology Study Team, Yazid Abdad, Celestine Aho, Christopher Blyth, Trevor Duke, Rebecca Ford, Andrew Greenhill, Paul Horwood, Ilomo Hwaihwanje, John Kave, Wendy Kirarock, Lea-Ann Kirkham, Tonny Kumani, Sarah Javati, Dorcas Joseph, Joe Jude, Amanda Lang, Deborah Lehmann, Geraldine Masiria, Barry Nagepu, Tilda Orami, William Pomat, Peter Richmond, Joycelyn Sapura, Peter Siba, Johanna Wapling, Mition Yoannes, Lapule Yuasi

**Affiliations:** 1 School of Paediatrics and Child Health, The University of Western Australia, Princess Margaret Hospital for Children, Roberts Road, Subiaco, 6008 WA Australia; 2 Wesfarmers Centre for Vaccines and Infectious Diseases, Telethon Kids Institute, University of Western Australia, PO Box 855, West Perth, 6872 WA Australia; 3 Department of Infectious Diseases and PathWest Department of Microbiology, Princess Margaret Hospital for Children, Roberts Road, Subiaco, 6008 WA Australia; 4 Papua New Guinea Institute of Medical Research, PO Box 60, Goroka, 441 Eastern Highlands Province Papua New Guinea; 5 School of Applied and Biomedical Sciences, Federation University Australia, Gippsland Campus, Northways Road, Churchill, 3842 VIC Australia; 6 Eastern Highlands Provincial Hospital, PO Box 392, Goroka, 441 Eastern Highlands Province Papua New Guinea

**Keywords:** Pneumonia, Meningitis, Papua New Guinea, *Streptococcus pneumoniae*, *Haemophilus influenza*e, Vaccination

## Abstract

**Background:**

Pneumonia and meningitis are common causes of severe childhood illness in Papua New Guinea (PNG). The etiology of both clinical conditions in PNG has not been recently assessed. Changes in lifestyle, provision and access to healthcare, antimicrobial utilization and resistance, and the national childhood vaccination schedule necessitate reassessment.

**Methods:**

A prospective case-control study was undertaken, enrolling children <5 years of age to determine the contemporary etiology of clinically defined moderate or severe pneumonia or suspected meningitis. Cases were identified following presentation for inpatient or outpatient care in Goroka town, the major population centre in the Eastern Highlands Province. Following enrolment, routine diagnostic specimens including blood, nasopharyngeal swabs, urine and (if required) cerebrospinal fluid, were obtained. Cases residing within one hour’s drive of Goroka were followed up, and recruitment of healthy contemporaneous controls was undertaken in the cases’ communities.

**Results:**

998 cases and 978 controls were enrolled over 3 years. This included 784 cases (78.6%) with moderate pneumonia, 187 (18.7%) with severe pneumonia and 75 (7.5%) with suspected meningitis, of whom 48 (4.8%) had concurrent pneumonia. The median age of cases was 7.8 months (Interquartile range [IQR] 3.9–14.3), significantly lower than community controls, which was 20.8 months (IQR 8.2–36.4). Half the cases were admitted to hospital (500/998; 50.1%). Recruitment of cases and controls and successful collection of diagnostic specimens improved throughout the study, with blood volume increasing and rates of blood culture contamination decreasing. The overall case fatality rate was 18/998 (1.8%). Of cases eligible for follow-up, outcome data was available from 76.7%. Low but increasing coverage of Haemophilus influenzae type B conjugate vaccines on the national schedule was observed during the study period: three dose DTPw-HepB-Hib coverage in children >3 months increased from 14.9 to 43.0% and 29.0 to 47.7% in cases and controls (both *p* < 0.001). Despite inclusion in the national immunization program in 2014, 2015 PCV13 three-dose coverage in cases and controls >3 months was only 4.0 and 6.5%.

**Conclusions:**

Recruitment of large numbers of pediatric pneumonia and meningitis cases and community controls in a third-world setting presents unique challenges. Successful enrolment of 998 cases and 978 controls with comprehensive clinical data, biological specimens and follow up was achieved. Increased vaccine coverage remains an ongoing health priority.

**Electronic supplementary material:**

The online version of this article (doi:10.1186/s41479-017-0029-y) contains supplementary material, which is available to authorized users.

## Background

Pneumonia and meningitis are common childhood diseases, resulting in significant morbidity and mortality among young children [1]. It is estimated that acute lower respiratory tract infection results in approximately 2.7 million deaths per annum, with 50% occurring in children <5 years of age. Of childhood pneumonia deaths, 33 and 16% are attributable to *Streptococcus pneumoniae* and *Haemophilus influenzae*, respectively [1]. Meningitis is estimated to result in >300,000 deaths (all ages) globally, with 26 and 21% estimated to be caused by *S. pneumoniae* and *H. influenzae* [1].

Papua New Guinea (PNG) was previously identified as a high priority country for the achievement of the United Nations Millennium Development Goals because the baseline child mortality rate is among the highest in the Western Pacific Region (<5 year old mortality rate in 2015: 57 per 1000 live births [2]). Pneumonia and meningitis are the most common causes for childhood deaths reported to the National Health Information Systems [3, 4]. Studies conducted in the Asaro Valley, including Goroka town, the provincial capital of Eastern Highlands Province (EHP), have repeatedly demonstrated the importance of *S. pneumoniae* and *H. influenzae* in the etiology of pneumonia and meningitis [5–11]. There are a paucity of contemporary data examining the bacterial causes of pneumonia and meningitis: the only recent study used opportunistically collected cerebrospinal fluid (CSF) samples from children admitted to Goroka General Hospital (GGH; 1996–2005) [9]. The current contribution of respiratory viruses to pneumonia also remains uncertain; apart from a single study assessing respiratory viruses in infants enrolled in a neonatal pneumococcal conjugate vaccine trial [12], prospective studies have not been undertaken since 1985 [13].

Conjugate *H. influenzae* type B (Hib) vaccine and 13-valent *S. pneumoniae* vaccine (13vPCV) were introduced into the national vaccination schedule in 2008 and 2014, respectively. From 2014, PNG infants were recommended neonatal BCG and hepatitis B vaccines followed by diphtheria, tetanus, whole-cell pertussis, hepatitis B and Hib vaccine (DTPw-HepB-Hib), 13vPCV and oral polio vaccines at 1, 2 and 3 months of age. Routine measles vaccines were recommended at 6 and 9 months [14]. In 2015, the PNG National Department of Health launched a Special Integrated Routine EPI Strengthening Program (SIREP Plus), a multimodal program aimed at improving coverage of vaccines already provided free under the national immunization program as well as introducing measles-rubella (MR, recommended at 6, 9 and 18 months) and inactivated polio vaccine (IPV, at 3 months) to all PNG children, and PCV13 to provinces yet to introduce the vaccine. Despite the 2014 launch, PCV13 was not available for use in children in the PNG highlands until 2015.

Given these changes, determining vaccine coverage in the PNG highlands and assessing the contemporary etiology of pneumonia and meningitis is a research priority. Commencing in 2013, we conducted a prospective case-control study enrolling children with clinically-defined moderate or severe pneumonia or suspected meningitis (cases) and contemporaneous community controls. This article describes the study methodology, population, vaccine coverage, and some of the operational and logistical challenges encountered throughout this study.

## Methods

We performed a prospective case-control study recruiting children with clinical pneumonia and/or suspected meningitis presenting either to the Eastern Highlands Provincial Hospital (EHPH, previously known as the Goroka General Hospital, GGH) or community healthcare clinics elsewhere in Goroka town and contemporaneous healthy community controls (January 2013 to January 2016). The date of commencement was chosen to ensure sufficient recruitment prior to the national introduction of the 13vPCV.

The study aims were to: (i) determine the etiology of pneumonia and meningitis in a setting with previously reported high rates of both diseases; (ii) identify the serotypes of *S. pneumoniae* causing invasive disease; (iii) determine the antimicrobial susceptibility profiles of isolated bacterial pathogens; (iv) examine the utility of molecular and other diagnostic assays in PNG children with pneumonia and meningitis and (v) explore clinical and laboratory variables associated with specific pathogens and severe diseases.

### Study location

The study was conducted in the EHP of PNG, an area of 11,157 km^2^, located at 6^0^30’S 145^0^40’E with a population of 579,825 (2011 census) [15]. Goroka, the provincial capital of EHP (population of 23,277) is located 1600 m above sea level, is only accessible by air from the national capital Port Moresby, and by a narrow mountainous highway to the nearest sea port, Lae. Sealed roads exist between major centres, but are often in poor condition. Feeder roads to villages are generally unsealed tracks and many villages and hamlets are only accessible on foot. Living standards vary significantly. In rural areas people live in hamlets in traditional thatched houses and are primarily subsistence farmers, but may obtain cash through sales of coffee and market produce. In the urban and peri-urban areas, many houses are built with strong and long-lasting materials such as an iron roof and cement walls and floors, although houses in the poorer settlements are more traditional and made of bush materials. Modern houses are connected to the town water and electricity supply (though interruptions are common) but this is less common in the settlements [16].

EHPH is the only tertiary healthcare facility in EHP and caters for all age groups. EHPH has a dedicated pediatric ward and children’s outpatient facility staffed by pediatricians, junior medical officers and pediatric nurses. In addition, urban clinics located in and around Goroka offer outpatient nurse-led care to children and adults. EHPH is the only site for inpatient hospital care in Goroka and surrounding villages and the EHPH children’s Outpatient Clinic, North Clinic and Lopi Clinic are the busiest outpatient facilities in the region.

Children presenting to the Children’s Outpatient Clinic, EHPH or two smaller urban clinics (North Clinic, Lopi Clinic) were eligible for recruitment. In addition, children presenting to the PNG Institute of Medical Research (PNGIMR) research clinic (located at the PNGIMR facility in Goroka, next to EHPH) or already admitted to the EHPH children’s ward were eligible for recruitment. Research was undertaken by PNGIMR staff.

### Recruitment of children with pneumonia and/or meningitis

Unwell children <5 years of age meeting the inclusion criteria were eligible for enrolment (see definitions of moderate and severe pneumonia and clinical meningitis as described in the PNG clinical manual and used nationwide [Table 1] [1]). Children not eligible for enrolment included those with severe congenital abnormalities, those hospitalized in the preceding 14 days and those previously enrolled within 30 days. Prior receipt of antibiotics was not an exclusion criteria. Eligible children were identified by clinically-trained research staff using a screening questionnaire. Upon determining eligibility and obtaining informed consent, research staff undertook a brief history and examination before laboratory specimens were obtained and treatment provided. Verbal consent and minimal data were obtained initially ensuring timely provision of appropriate medical care. This was followed by a more in-depth interview exploring risk factors for disease, recent antibiotic exposure, vaccination history and symptoms, examination including oximetry (recorded using LifeBox Pulse Oximeter, Acare Technology Co. Ltd, Taiwan), written consent and further specimen collection as required. In addition to parental interview, individual health records were examined for perinatal history, co-morbidities and vaccination status.

**Table 1:** Inclusion and exclusion criteria including clinical case definitions [14]

Criteria	Classification	Definitions
Inclusion Criteria (cases)	Moderate pneumonia	Presence of all three symptoms and signs: • cough • tachypnoea rate (≥60/min if <2 months, ≥40/min if ≥2 mo of age) • lower chest wall indrawing
	Severe pneumonia	Moderate pneumonia with one of the following added features: • pulse >160/min with hepatomegaly >2 cm below costal margin • cyanosed or restless • inability to breastfeed/drink or vomiting.
	Suspected meningitis	Defined by the presence of fever (>37.5 °C) and one of the following additional features: • seizures • impaired or altered consciousness • irritability • a bulging fontanelle • signs of meningeal irritation (e.g. neck stiffness, Kernig’s sign).
Exclusion criteria (cases)		The following children were excluded from enrolment as cases: • age ≥ 5 years • severe congenital abnormality • hospitalisation in preceding 14 days • previous enrolment as either case or control in the study within 30 days • parent unable or unwilling to give consent
Exclusion criteria (controls)		The following children were excluded from enrolment as controls • age ≥ 5 years • symptoms or signs of active infection including cough, tachypnoea, lower chest wall indrawing, fever (>37.5 °C), altered consciousness, drowsy or “staring eyes”, irritability, bulging fontanelle and neck stiffness or signs of meningeal irritation • severe congenital abnormality • hospitalization in preceding 14 days • previous enrolment in the study as a control^a^ • exposed to antibiotics in the past 30 days • parent unable or unwilling to give consent

*Abbreviations*: *Mo* months

^a^ Controls could be subsequently enrolled as cases if more than 30 days since previous enrolment

Children meeting the inclusion criteria yet deemed well enough for outpatient management were still eligible for enrolment. In addition to children enrolled through outpatient clinics, inpatients presenting after-hours were enrolled following parental consent. This was restricted during the first year of the study to those identified <48 hours following admission, but was extended to 96 h to enhance recruitment, particularly for children with suspected meningitis perceived to be too sick to undergo an early lumbar puncture.

All admitted cases were reassessed daily during their hospital stay by clinically trained research staff. Children deemed well enough to be managed as outpatients were discharged and reviewed within 48 h. Contact mobile phone numbers, if available, were collected from all parents prior to discharge. Review of all children residing within one hour (by vehicle and/or foot) from Goroka was attempted, 2 to 4 weeks post-discharge. At review, their health status was assessed and the presence of ongoing symptoms and/or signs confirmed.

### Recruitment of controls

Healthy children <5 years of age were enrolled to assist in determining community vaccination coverage and for further nasopharyngeal bacterial colonization and viral studies. Children were eligible if they met the inclusion/exclusion criteria (Table 1) and were recruited during community follow-up visits for discharged cases. PNGIMR staff conducted community clinics to provide primary healthcare and vaccination, thereby facilitating further identification and recruitment of controls. Given the logistical challenges of recruiting matched controls, formal case-control matching was not undertaken although contemporaneous controls were recruited from the same communities as cases. In an attempt to reduce bias, the number of cases recruited and median age were regularly assessed and used to guide targeted control recruitment. Following informed consent, a brief history and examination was undertaken, individual health and immunization records were examined and two nasopharyngeal swabs were collected.

### Specimen collection

Consent was sought to collect blood, nasopharyngeal specimens, urine and (if clinically indicated) CSF from all cases (Additional file 1: Table S1), and nasopharyngeal specimens from controls. Although recommended in cases of suspected meningitis, a decision to collect CSF was left to the treating pediatric staff. Using appropriate aseptic technique (iodine solution followed by 70% alcohol, ensuring adequate drying time prior to procedure), PNGIMR research staff collected up to 5 ml of whole blood for culture (inoculated directly into BD BACTEC™ PedsPlus bottles; BD, Franklin Lakes, New Jersey, United States [US]), PCR and antimicrobial activity. Blood volume was recorded by clinical staff prior to blood culture inoculation. Additional blood tests, if clinically indicated (e.g. malaria blood slides, haemoglobin, human immunodeficiency virus [HIV] serology) were also obtained. If obtainable, urine was collected for antimicrobial activity and storage. Two deep-flocked nasopharyngeal swabs (Copan Diagnostics, Murrieta, California, US) were collected on all children: the first placed immediately in 1 ml of skim milk tryptone glucose glycerol broth (STGGB) and the second in 1 ml of viral transport media (VTM). Following initial processing, STGGB samples were stored at −80 °C whilst EDTA, serum, urine and VTM samples were stored at −20 °C. Blood and CSF culture contamination rates were monitored throughout the study and a review of collection technique and additional staff training was undertaken regularly during periods of higher-than-expected contamination.

### Laboratory processing

All laboratory testing was undertaken at the PNGIMR Bacteriology and Molecular Laboratories, Goroka. Blood cultures were incubated for up to 5 days at 35 °C using a BD BACTEC™ automated blood culture system (BD, Franklin Lakes, NJ) or when unavailable, a 35–37 °C incubator. Positive bottles (using the BACTEC system) were subcultured onto standard and selective media and incubated with/without 5% CO_2_. During periods when the BACTEC system was unavailable, manual daily subculture from inoculated BACTEC™ bottles was undertaken using methods previously described [5]. All CSF underwent macroscopic examination, manual cell count and Gram stain. In the setting of CSF pleocytosis, CSF was cultured on standard media and incubated with/without 5% CO_2_, bacterial antigen detection for S.*pneumoniae* and *H.influenza*e using latex agglutination was performed and further staining for acid-fast bacilli and yeast undertaken. CSF biochemistry (protein and glucose) was undertaken using Uristix® reagent urinalysis strips (Siemens, Victoria, Australia).

Bacterial isolates were identified using colony morphology and standard confirmatory tests (*S.pneumoniae*: optochin sensitivity, bile solubility, catalase; *H.influenzae*: differential growth on blood/chocolate agar, X&V disks). Serotyping was undertaken by the Quellung reaction using commercially obtained antisera (Staten Serum Institut, Denmark) and *H.influenzae* agglutinating sera (Remel, Dartford, United Kingdom). Antimicrobial susceptibility testing was conducted on all clinically relevant isolates by disc diffusion with suspected resistance confirmed by E-tests® (bioMérieux, Durham, North Carolina, US) using Clinical and Laboratory Standards Institute (CLSI) breakpoints [17].

For real-time PCR on blood and CSF samples, DNA was extracted from 200 μl blood/CSF using commercially available kits (Qiagen DNease Blood & Tissue Kit, Valencia, California, US) and an in-house real-time PCR for *S.pneumoniae* (*lyt*A) and *H. influenzae (hpd*) performed [18, 19]. In general, replicate samples within 0.5 Cq of each other and with a Cq value ≤35 (but within the limit of detection as determined by the standards) were deemed to be positive.

Respiratory virus testing was undertaken using the dedicated VTM sample and an in-house multiplexed real-time quantitative PCR assay. DNA/RNA were extracted using commercially available kits (Qiagen DNease Blood & Tissue Kit, Valencia, California, US). Viral targets are described in Additional file 2: Table S2 [20–22]. Semi-quantitative bacterial culture of STGGB was performed using blood, chocolate, gentamicin-blood and bacitracin-chocolate agar plates, incubated with 5% CO_2_. Bacterial isolates were identified and typed and susceptibility testing performed as described above.

Antibiotic activity was measured in blood, urine and/or CSF to better understand the yield of blood and CSF cultures. This was performed by placing a specimen-soaked filter paper disk on agar inoculated with a 0.5 MacFarland suspension of *Staphylococcus aureus* ATCC 25923. Following incubation at 37 °C overnight, a zone of inhibition was examined and measured.

### Radiology

When a chest X-ray was requested as part of clinical care, digital copies were extracted and stored on a dedicated hard drive. These will be reviewed by international experts using standardised methods [23].

### Ethics, data and study management and analysis

Ethical approval was obtained from the Institutional Review Board of PNGIMR (IRB no 1204), the Medical Research Advisory Committee of PNG (MRAC number 11.29) and the University of Western Australia (RA/4/1/7960). Clinical and laboratory data were collected on paper forms, subsequently checked by a second staff member and entered in a dedicated Filemakerpro database 11 (DB services, Indianapolis, IN, US). Monthly teleconferences and regular data checks were undertaken to ensure the accuracy of all data collected.

Analysis was undertaken using SPSS 20.0.0 (IBM Corp, New York, NY, US). Continuous variables were compared with the Student’s *t* test, and categorical variables were compared with *χ*2 or Fisher’s Exact test. A *p* value of ≤0.05 was considered significant.

## Results

From January 2013 to January 2016, 1001 children with pneumonia or meningitis and 994 heathy children were consented to the study. Three cases and 16 controls were excluded from the analysis as they did not meet the inclusion criteria (cases: not meeting clinical case definitions [*n* = 2]; >5 years of age [*n* = 1]; controls: evidence of acute respiratory infection [*n* = 5]; >5 years of age [*n* = 11], Table 1). Thirty-four children were enrolled as cases more than once and 14 controls were subsequently enrolled as cases.

### Pneumonia and meningitis cases

The median age of enrolled cases was 7.8 months (interquartile range [IQR] 3.9–14.3) and 562/998 cases were male (56.3%; Table 2). Parents infrequently reported chronic health concerns (12/984; 1.2%), the most frequent being chronic respiratory disease (*n* = 6) and seizures (*n* = 4). Ninety-six (9.6%) and 7 (0.7%) cases reported a history of pneumonia and meningitis, respectively.

**Table 2:** Cases and controls: demographics, recruitment, diagnosis, diagnostic specimens and follow up

	Cases: *n* (%)(*n* = 998)	Controls: *n* (%)(*n* = 978)
Demographics		
Median age (months; IQR)	7.8 months (3.9–14.3)	20.8 months (8.2–36.4)
Male sex (%)	562 (56.3%)	520 (53.2%)
Recruitment year		
2013	286 (28.6%)	172 (17.6%)
2014	291 (29.1%)	288 (29.4%)
2015^a^	421 (42.2%)	518 (53.0%)
Recruitment site		
Children’s ward, EHPH	404 (40.5%)	NA
Outpatient clinics, EHPH	313 (31.4%)	
North Clinic	194 (19.4%)	
Lopi Clinic	7 (0.7%)	
PNGIMR Research Clinic	75 (7.5%)	
Unknown	5 (0.5%)	
Diagnosis		
Moderate pneumonia • Without suspected meningitis • With suspected meningitis	784 (78.6%) 753 31	NA
Severe pneumonia • Without suspected meningitis • With suspected meningitis	187 (18.7%) 170 17	
Suspected Meningitis (without pneumonia)	27 (2.7%)	
Diagnostic specimens collected		
Blood culture	987 (98.9%)	NA
EDTA blood	512 (51.3%)	NA
Serum	363 (36.4%)	NA
CSF	31 (3.12%)	NA
Nasopharyngeal swab	998 (100%)	978 (100%)
Urine	42 (4.2%)	NA
Successful follow up (of eligible children) by year		
2013	156/223^b^ (69.9%)	NA
2014	162/209^b^ (77.5%)	
2015^a^	251/310^b^ (81.0%)	

*Abbreviations*: *IQR* interquartile range, *EHPH* Eastern Highlands Provincial Hospital, *NA* not applicable

^a^ inclusive of the first 3 weeks of January 2016

^b^ denominator is children residing within 1 h by vehicle from Goroka

**Fig. 1:**
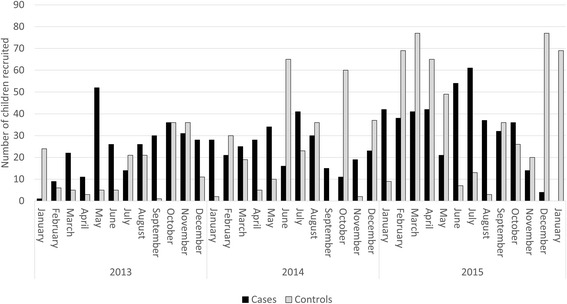
Enrolment of cases and controls by month: January 2013 to January 2016

Case enrolment during the study increased from 286 cases in 2013 to 421 in 2015 (Table 2). Despite the seasonal nature of pneumonia (peak activity during the southern hemisphere winter), cases were recruited year round: the average number of cases recruited per month was 36 (range 9–61; Fig. 1). A significant proportion of cases were recruited following admission to EHPH (404/998; 40.5%) with the remainder recruited from the EHPH outpatient clinic (313; 31.4%), community (201; 20.1%) or PNGIMR research clinics (75; 7.5%). The proportion of children recruited prior to admission to hospital (i.e. from EHPH outpatients or community clinics, as opposed to the EHPH children’s ward) increased over the duration of the study. This shift in recruitment was part of a dedicated focus to enhance recruitment of children prior to antibiotic delivery (2013: 30.8% recruited prior to admission to EHPH, 2014: 58.1%, 2015: 80.1%; *p* < 0.0001).

The most frequent diagnosis was moderate pneumonia (*n* = 784, 78.6%), followed by severe pneumonia (*n* = 187, 18.7%) and suspected meningitis (with or without pneumonia; *n* = 75, 7.5%). Nasopharyngeal samples were collected on all cases with blood obtainable from 987 children (98.9%; Table 2). The volume of blood collected per patient increased throughout the study period: mean volume in 2013, 1.36 ml; 2014, 1.49 ml; 2015, 1.95 ml; difference in means; 0.12 ml, (95% confidence interval [CI]: 0.016–0.224; *p* <0.03) and 0.46 ml (95%CI: 0.32–0.61; *p* < 0.0001), respectively. In addition, the number of children with dedicated EDTA samples increased over time: 2013, 89/286 (31.1%); 2014, 150/291 (51.5%) and 2015, 273/421 (64.8%), *p* < 0.001. The rate of contaminated blood cultures decreased during the study: 2013, 63/281 (22.4%); 2014, 41/285 (14.4%) and 2015, 48/421 (11.4%), *p* < 0.001. Collection of blood prior to antibiotics was also more frequently achieved over the course of the study: 2013, 139/275 (50.5%); 2014, 198/282 (70.2%) and 2015, 246/415 (83.4%), *p* < 0.001.

Both CSF and urine samples were infrequently collected during the study. In children with a clinical diagnosis of suspected meningitis (*n* = 75), only 29 underwent a CSF examination. Of the remainder, 24 cases were deemed by EHPH medical staff not to have clinical evidence of meningitis, seven cases had contraindication to lumbar puncture and eight had a lumbar puncture without CSF being obtained. In a further seven cases with suspected meningitis, it is uncertain why sampling was not performed. A further 2 CSF samples were taken from children not meeting the clinical definition of meningitis.

Half the patients were admitted to hospital (500/998; 50.1%) with the remainder managed as outpatients. Less than 25% of children had an X-ray performed. The median length of hospital stay was 2 days (IQR 2–5).

Eighteen deaths were identified (overall case fatality rate (CRF): 18/998, 1.8% overall; pneumonia, 1.5%; meningitis, 8.0%). The in-hospital CFR was 14/500 (2.8% overall: pneumonia, 2.3%; meningitis 8.7%). Follow-up was attempted in children residing within 1 h by vehicle from Goroka (*n* = 742). Outcome data was obtained on 569 children (76.7%). During the period of the study, the proportion of eligible children that were successfully followed up increased from 69.9% (2013) to 81.0% (2015), *p* < 0.02.

### Controls

The median age of enrolled controls was 20.8 months (IQR 8.2–36.4) and 522/983 enrolled controls were male (53.2%; Table 2). Successful recruitment of controls increased during the study period, particularly following the institution of dedicated clinics in 2014 and 2015. Despite ongoing attempts to recruit younger controls, controls remained significantly older than cases throughout the study; median ages cases vs. controls: 6.6 vs. 14.3 months (2013), 9.3 vs. 22.7 months (2014) and 8.4 vs. 21.0 months (2015).

### Vaccination coverage

**Fig. 2:**
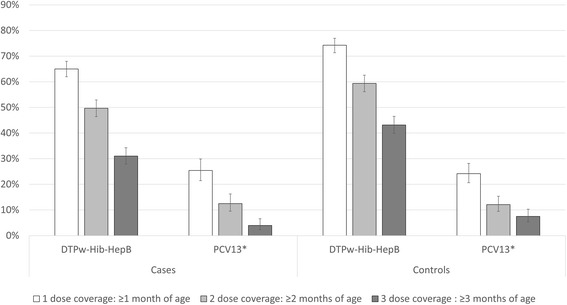
Vaccine coverage by case-control status and age (*PCV13 data for 2015 only)

Child health books that included vaccination status were assessable in 991 cases (99.3%) and 976 controls (99.8%). Coverage for one dose of DTPw-HepB-Hib in children >1 month of age was 65.5% in cases and 71.9% in controls (*p* < 0.01, Fig. 2), falling for three doses in children >3 months of age to 31.6% in cases and 43.3% in controls (*p* < 0.001). The significant difference in vaccine coverage between cases and controls remained when restricted to children < 12 months of age at enrolment. The overall median age of the first, second and third dose of DTPw-HepB-Hib was 1.4 months (IQR 1.1–3.0), 3.1 months (IQR 2.1–6.1) and 4.7 months (IQR 3.1–8.0). During the study, vaccine coverage significantly improved: three dose DTPw-HepB-Hib coverage in children >3 months of age increased from 14.9 to 43.0% in cases and 29.0 to 47.7% in controls (*p* < 0.001 in both).

Despite the launch of the PCV13 program in 2014, the vaccine was not available in the EHP until 2015. The 2015 coverage of PCV13 was low in both cases and controls with only 25.4 and 24.4% of cases and controls >1 month receiving one dose, and 4.0 and 6.5% of cases and controls >3 months receiving three doses (no significant difference between cases and controls).

## Discussion

The ongoing burden of childhood disease in PNG necessitates a contemporary reassessment of pneumonia and meningitis etiology and outcomes. Given the lack of access to and (if present) underutilisation of the microbiological laboratory in PNG, targeted projects using routine and contemporary diagnostics are required to assess the current pathogen-specific burden of disease. These data are essential to assess potential benefits of childhood vaccination and to identify potential changes in etiology.

Undertaking such studies, particularly in locations such as the highlands of PNG, pose unique challenges in study design, recruitment, specimen handling and laboratory practice. A number of these challenges are unique to both PNG and to other isolated highland communities, while others are frequent with any large prospective study. This paper outlines the methods and challenges observed in recruiting nearly 2000 children over 3 years.

Based on existing EHPH service data, the study proposed to recruit >1000 children with clinical pneumonia and meningitis over 2 years. Prior to commencement, concern was raised by the community about the invasive nature of the specimens proposed. PNGIMR has a long history of community engagement developed over many successful research studies. Regular visits to communities to discuss this study, explaining the routine nature of specimen collection in other healthcare settings and the collaborative nature of healthcare provided by PNGIMR and hospital staff was essential in the early phase of the study. The provision of non-monetary incentives such as transport from hospital to home for study participants and future access to the PNGIMR clinic are also likely to have assisted with recruitment, which improved throughout the study.

Barriers to recruitment included frequent and sometimes prolonged industrial action leading to closure of health facilities. As a result, lower numbers of children were admitted to the ward and at times, PNGIMR staff needed to focus on providing healthcare rather than the research project. Outbreaks of vaccine preventable diseases (e.g. an outbreak of measles in April to June 2015) required additional healthcare resources to be diverted away from research activities. Hospital senior and junior medical staff changed several times during the 3-year study period and a lack of familiarity with the study required regular re-engagement by the research team with the EHPH paediatric staff. Significant variation in practice, especially with CSF collection and utilisation of radiology were noted throughout the study. As both procedures were not mandated, rather recommended based on clinical need, it is likely that physician preference contributed to variable utilisation. Provision of a dedicated study doctor may have assisted with the variation in practice observed.

Compared with previous pneumonia studies conducted at EHPH [8], a number of differences should be noted: a lower proportion of enrolled children had severe pneumonia (19% of the pneumonia cohort in this study vs 46% in Barker et al. [8]). A higher number were deemed well enough to manage as outpatient following initial treatment (50% vs 0% in Barker et al. [8]). Forty per cent of children were enrolled from the ward following initial management by EHPH staff, albeit in decreasing numbers over the duration of the study. A significant number of children had been administered antibiotics even prior to their first interview at EHPH (data not shown). It is expected that these factors will reduce the rate of detection of bacteraemia and invasive bacterial isolates from the current cohort. The in-hospital mortality rate (2.8%) is significantly lower than Barker et al. (8%) [8] and the rate reported nationally (5.2%) by the Paediatric Hospital Reporting (PHR) system, yet comparable to 2013 PHR data collected from EHPH (2.4%) [4]. These severity of illness and mortality data are encouraging and suggest that, compared with past studies, improvements in access to health services, earlier recognition of childhood pneumonia with earlier institution of antimicrobial therapy and supportive care are leading to better outcomes for children with pneumonia.

Recruitment of controls was challenging, particularly in the early phases of the study. Despite regular visits to rural and peri-urban communities, well children (particularly those < 6 months of age) were often absent, frequently accompanying their parents to the fields early in the morning. There was an attempt to overcome this by scheduling PNGIMR community clinics and establishing age specified targets. Following institution of these clinics, larger numbers of controls were recruited, yet recruitment of younger controls remained an ongoing challenge. Given the uneven balance between cases and controls, assessment of biological samples (e.g. nasopharyngeal samples) will be undertaken in cases and controls matched post-enrolment by month of illness, age and village.

PNG has a long history of utilization of a hand-held child health record. Documentation of vaccination status is routinely recorded in the health book (on the reverse of front cover and in the continuous clinical records). The health record was assessable in more than 99% of cases and controls. The variable nature of recording required additional training and care to ensure accuracy of the data collected by the research team. This may have resulted in underestimation of the vaccine coverage early in the study. Replacement of lost hand-held records or an older child having more than one book may result in underestimation of vaccine uptake (data not presented). Despite this, the record provides a unique opportunity to assess uptake of vaccination status in a country where access to electronic or manually collected vaccine distribution data remains a challenge. Vaccine coverage in the highlands remains lower than the reported national average [2]: the reasons for this are likely to be multifactorial and may include reduced access to health services given the remoteness of highland villages, problems with consistent immunization supply and parental education with ongoing mistrust of infant immunization. Increased vaccine coverage is encouraging and suggests that targeted programs including the Special Integrated Routine EPI Strengthening Program (SIREP) is having an impact in the PNG highlands.

Undertaking a study dependent on key infrastructure in isolated locations poses unique challenges. For much of the study the BD BACTEC™ Instrumented Blood Culture System was not working, despite multiple attempts by the laboratory staff and the manufacturer to repair the machine both in Goroka and Port Moresby. The laboratory was reliant on manual subculture of blood cultures increasing the time and risk of contamination of critical specimens. Higher rates of contamination are likely to impact on the ability to detect significant bacteraemia. For significant periods of time, the hospital X-ray facility required repairs, meaning that physician-requested X-rays were not available. These key challenges must be considered when designing studies in third-world settings, particularly in locations outside the nation’s capital city with difficult access by road or by air.

Achieving a follow up rate of >75% in an environment where formal town maps and permanent phone connections are largely non-existent is a testament to both study participants and staff. This was achieved by meticulous documentation of village, hamlet and house location and use of PNGIMR drivers to return children and their parents to their homes. This was also enhanced by the provision of ongoing healthcare clinics in larger villages and access for study participants to the PNGIMR clinic. Despite these incentives, numerous attempts were often required for follow up.

## Conclusions

In summary, recruitment of large numbers of pediatric pneumonia and meningitis cases and community controls in a third-world setting with limited health services, health and other infrastructures presents unique challenges. The study successfully enrolled 998 cases and 978 controls, obtained comprehensive clinical data and biological specimens, and was able to follow up >75% following discharge. Furthermore, staff documented increasing coverage of pneumococcal and Hib conjugate vaccines in PNG children. Key aspects to overcoming the challenges of conducting research in third-world settings include a pragmatic and flexible study design and strong collaborative relationships with healthcare providers. Prior to commencing research, the infrastructure needs of the study (and alternative plans should key equipment be unavailable) must be considered. Ongoing community engagement is paramount to optimize recruitment and translation. In addition, the provision of ongoing training for both research and clinical staff ensures staff retention and facilitates translation of research findings into changes in clinical practice.

## Additional files

Additional file 1: Table S1. Specimens collected and laboratory tests performed. (DOC 35 kb)

Additional file 2: Table S2. Nasopharyngeal specimens collected into viral transport media: Gene targets for viruses and bacteria [20–22]. (DOC 44 kb)

## Abbreviations

13vPCV: 13-valent Pneumococcal conjugate vaccine
CSF: Cerebrospinal fluid
EHP: Eastern Highlands Province
EHPH: Eastern Highlands Provincial Hospital
GGH: Goroka General Hospital
HIV: Human immunodeficiency virus
PCR: Polymerase chain reaction
PHR: Paediatric Hospital Reporting
PNG: Papua New Guinea
PNGIMR: Papua New Guinea Institute of Medical Research
STGGB: Skim milk tryptone glucose glycerol broth
VTM: Viral transport media
