# Supplementary material for: Childhood pneumonia and meningitis in the Eastern Highlands Province, Papua New Guinea in the era of conjugate vaccines: study methods and challenges
Source: Pneumonia (Nathan). 2017 Mar 5;9:5. doi: 10.1186/s41479-017-0029-y (PMC5471671; doi:10.1186/s41479-017-0029-y)
Supplement: Supplementary file 1 — Specimens collected and laboratory tests performed. (DOC 35 kb) [file 41479_2017_29_MOESM1_ESM.doc]

Supplemental Table 1: Specimens collected and laboratory tests performed

| **Sample** | **Collection details** | **Sample used for** |
| --- | --- | --- |
| **Blood** | Up to 5ml  (maximum of 3.5ml in children < 6 months or deemed to be severely unwell) | - Bactec® Blood culture bottle for culture, identification and susceptibility testing (2-3ml) - EDTA blood for DNA/RNA isolation followed by *S. pneumoniae* and *H. influenzae* PCR - Antibacterial activity: - Serum for storage, future testing and assay development |
| **Nasopharyngeal swab** | Swab inserted into the nasopharynx until resistance felt and then rotated slowly for 5 seconds | - Bacterial culture: 1ml of Skim-Milk-Tryptone-Glucose-Glycerol-Broth for Culture for *S. pneumoniae, H.H. influenzae* and other respiratory bacterial pathogens - Viral culture and PCR: 1ml of viral transport media for viral culture and quantitative real-time PCR for respiratory viruses |
| **Urine** | Collected by clean-catch or urine bag | - Antibacterial activity |
| **Cerebrospinal fluid** | Up to 3ml  (maximum of 1.5ml in children < 6 months or deemed to be severely unwell) | - Cell count, Gram stain, Culture (and susceptibility testing) - DNA/RNA isolation and *S. pneumoniae* and *H. influenzae* PCR - Antibacterial activity - Additional tests if clinically indicated - Stored CSF for future testing and assay development |
