# Supplementary material for: Childhood pneumonia and meningitis in the Eastern Highlands Province, Papua New Guinea in the era of conjugate vaccines: study methods and challenges
Source: Pneumonia (Nathan). 2017 Mar 5;9:5. doi: 10.1186/s41479-017-0029-y (PMC5471671; doi:10.1186/s41479-017-0029-y)
Supplement: Supplementary file 2 — Nasopharyngeal specimens collected into viral transport media: Gene targets for viruses and bacteria [20–22]. (DOC 44 kb) [file 41479_2017_29_MOESM2_ESM.doc]

Supplemental Table 2. Nasopharyngeal specimens collected into viral transport media: Gene targets for viruses and bacteria [20-22]

| **Pathogen** | **Gene target** |
| --- | --- |
| Respiratory Syncytial Virus (RSV) | Matrix (M) protein coding region |
| Parainfluenza type 1 | Matrix (M) protein coding region |
| Parainfluenza type 2 | Nucleoprotein (N) coding region |
| Parainfluenza type 3 | Nucleoprotein (N) coding region |
| Human metapneumovirus | Nucleoprotein (N) coding region |
| Human rhinovirus | 5’ Untranslated region |
| Human coronaviruses | Nucleoprotein (HCoV 229E, OC43, NL63) and replicase polyprotein 1ab (HcoV-HKU1) coding region |
| Human adenovirus | Hexon coding region |
| Bordatella pertussis | Insertion sequence 481 |
